# Postoperative adjuvant radiotherapy is associated with improved survival in hepatocellular carcinoma with microvascular invasion

**DOI:** 10.18632/oncotarget.20402

**Published:** 2017-08-23

**Authors:** Liming Wang, Weihu Wang, Xuesong Yao, Weiqi Rong, Fan Wu, Bo Chen, Mei Liu, Shengtao Lin, Yunhe Liu, Jianxiong Wu

**Affiliations:** ^1^ Department of Hepatobiliary Surgery, National Cancer Center/Cancer Hospital, Chinese Academy of Medical Sciences and Peking Union Medical College, Beijing, China; ^2^ Department of Radiation Oncology, Key Laboratory of Carcinogenesis and Translational Research (Ministry of Education/Beijing), Peking University Cancer Hospital and Institute, Beijing, China; ^3^ Department of Interventional Therapy, National Cancer Center/Cancer Hospital, Chinese Academy of Medical Sciences and Peking Union Medical College, Beijing, China; ^4^ Department of Radiation Oncology, National Cancer Center/Cancer Hospital, Chinese Academy of Medical Sciences and Peking Union Medical College, Beijing, China; ^5^ Laboratory of Cell and Molecular Biology and State Key Laboratory of Molecular Oncology, National Cancer Center/Cancer Hospital, Chinese Academy of Medical Sciences and Peking Union Medical College, Beijing, China

**Keywords:** hepatocellular carcinoma, microvascular invasion, TACE, radiotherapy, relapse-free survival

## Abstract

**Purpose:**

Limited studies have compared the efficacy of postoperative adjuvant therapies in HCC patients with microvascular invasion (MVI). In this study we assess the efficacy of postoperative adjuvant conservative therapy (CT), trans-catheter arterial chemoembolization (TACE) and radiotherapy (RT) in HCC patients with MVI.

**Results:**

Kaplan-Meier survival analysis revealed that patients in the RT group have significantly improved RFS (RT vs TACE: *p* = 0.011; RT vs CT: *p* < 0.001) and OS (RT vs. TACE: *p* = 0.034; RT vs CT: *P* < 0.001) compared to TACE and CT groups. Further, subgroup analysis based on the degree of MVI and surgical margin width showed that patients with narrow surgical margin have significantly longer RFS and OS after adjuvant RT than the TACE and CT, independent of degree of MVI. Multivariate analysis indicated that MVI classification is the independent prognostic factor associated with RFS and OS.

**Materials and Methods:**

Between July 2008 and December 2015, 136 HCC patients with MVI were divided into three groups according to their adjuvant therapies. Survival outcomes namely relapse-free survival (RFS) and overall survival (OS) of the three groups were analyzed.

**Conclusions:**

Adjuvant radiotherapy following hepatectomy could result in better survival outcomes for HCC patients with MVI than TACE or CT.

## INTRODUCTION

Hepatocellular carcinoma (HCC), the primary malignancy of the liver, is the sixth most common cancer and the second leading cause of cancer-related mortality in the world [1]. It is of major concern in less developed countries where it accounts for 83% of the estimated 782,000 new cancer cases [1, 2]. Eastern Asia and sub-Saharan Africa reported the highest age adjusted incidence rate of over 20 per 100,000 individuals [3]. In China, primary liver cancer is the second most common cancer and accounts for 55% of all HCC cases, [2] and nearly 383,203 persons die of liver cancer every year and this accounts for 51% of liver cancer deaths worldwide [4]. Cirrhosis caused by hepatitis B and hepatitis C virus infections is the main risk factor for HCC [2–4]. Surgical resection and liver transplantation are the standard of care curative treatment modalities for HCC in cirrhotic patients with good functional reserves [5]. However, postoperative intrahepatic recurrence of HCC remains a significant clinical problem with recurrence rate as high as 70%-100% at 5-years after resection and 15–30% after liver transplantation [6, 7]. The various risk factors for recurrence of HCC include tumor size, tumor number, vascular invasion (both macroscopic and microscopic), presence of stellate nodules, histopathological grade, underlying cirrhosis and the type of surgery (i.e. narrow vs. wide surgical margins, anatomic vs. non-anatomic resection, minor vs. major resections) [6].

Microvascular invasion (MVI), is the most important risk factor that is significantly associated with early postoperative recurrence in the liver remnant and is confirmed as an independent predictor of both overall and disease-free survival after liver resection [7–9]. Even for patients with small HCCs, MVI increase the rate of tumor recurrence and dramatically shortens long-term survival [10, 11]. Unfortunately, MVI can be detected only by postoperative histological examination, which makes the postoperative treatment an essential requirement.

The effectiveness of adjuvant radiotherapy in reducing the recurrence and improving the overall survival is well documented in clinical studies [12, 13]. Earlier study has shown that postoperative intensity-modulated radiation therapy (IMRT) improved 3-year overall and disease-free survival in HCC patients receiving narrow margin hepatectomy close to major vessels [12]. In addition, the post-hoc subgroup comparison of randomized trial also demonstrated that adjuvant radiotherapy improved recurrence-free survival in patients with small HCC lesions [13]. Although, various adjuvant therapies such as TACE, radiation therapy, interferon, polyprenoic acid, adoptive immunotherapy and iodine-131-labeled lipiodol were used to decrease the recurrence and prolong the survival; it is important to note that studies comparing these adjuvant therapies taking the MVI into consideration are scarce. Consequently, the optimal postoperative treatment algorithms for preventing recurrence of HCC in patients with MVI have not been thoroughly evaluated. Therefore, in this retrospective, single-center study, we analyze the survival outcomes of HCC patients with MVI who received conservative treatment, radiotherapy or TACE as their primary adjuvant treatment after hepatectomy and analyzed the variables influencing the outcomes in these patients.

## RESULTS

### Demographic and clinicopathological characteristics

A total of 136 patients (118 male, 18 female) with a mean age of 53.51 ± 11.36 years (range 27–79) were included in the study (CT group, *N* = 50; TACE group, *N* = 42; RT group, *N* = 44). The baseline demographic and the clinicopathological characteristics of the three groups of patients with MVI are summarized in Table 1. Most of the characteristics of the three groups were comparable. Patients in the CT group were significantly older (*p* = 0.014) and those in RT group had significantly higher rates of narrow surgical margin (*p* = 0.010) than the other two groups.

**Table 1:** The baseline characteristics and demographics of patients in the CT, TACE and RT groups

Variable	Postoperative therapeutic management			*p* value
	CT (*n =* 50)	TACE (*n =* 42)	RT (*n =* 44)	
Age (mean ± SD)	57.22 ± 11.14	51.38 ± 10.89	51.32 ± 11.21	0.014
Gender (Male/Female)	5/45	8/34	5/39	0.401
Operative time (min)	210.84 ± 71.88	217.38 ± 81.56	238.93 ± 76.34	0.188
Operative procedure (Major/Minor)	33/17	24/18	26/18	0.652
Operative method (Anatomical/Non-anatomical)	27/23	21/21	23/21	0.929
Blood loss (ml)	516.00 ± 451.35	506.67 ± 493.24	632.96 ± 478.00	0.378
Surgical margin (≤ 1 cm/> 1 cm)	29/21	22/20	36/8	0.010
Tumor size (cm)	5.71 ± 2.60	6.15 ± 3.65	5.63 ± 2.73	0.693
Number of tumor (single/multiple)	46/4	38/4	41/3	0.899
Differentiation (Well/Moderate/Poorly)	2/30/18	1/24/17	1/24/19	0.423
MVI classification (M1/M2)	36/14	24/18	32/12	0.216
Envelope invasion (Present/Absent)	9/41	8/34	13/31	0.344
Cirrhosis (Present/Absent)	2/48	4/38	4/40	0.336
Viral hepatitis (Negative/Positive)	2/48	0/42	1/43	0.543
Preoperative AFP level (≤ 400 ng/L/> 400 ng/L)	32/18	25/17	34/10	0.186
Preoperative ALT (U/L)	34.40 ± 22.86	34.14 ± 24.28	41.86 ± 31.32	0.293
Preoperative TBIL(µmol/L)	12.50 ± 5.97	12.55 ± 5.23	12.55 ± 4.67	0.998
Preoperative ALB (g/L)	40.91 ± 4.78	42.87 ± 5.47	42.43 ± 4.20	0.121
Preoperative PALB (mg/dL)	18.16 ± 5.49	19.43 ± 5.72	20.30 ± 5.55	0.178
Preoperative PTa (%)	83.97 ± 10.70	84.41 ± 10.31	80.97 ± 9.53	0.230
Preoperative Child-Pugh (A/B)	50/0	42/0	44/0	-

### Relapse-free survival and overall survival rates of the CT, TACE and RT groups

**Figure 1:**
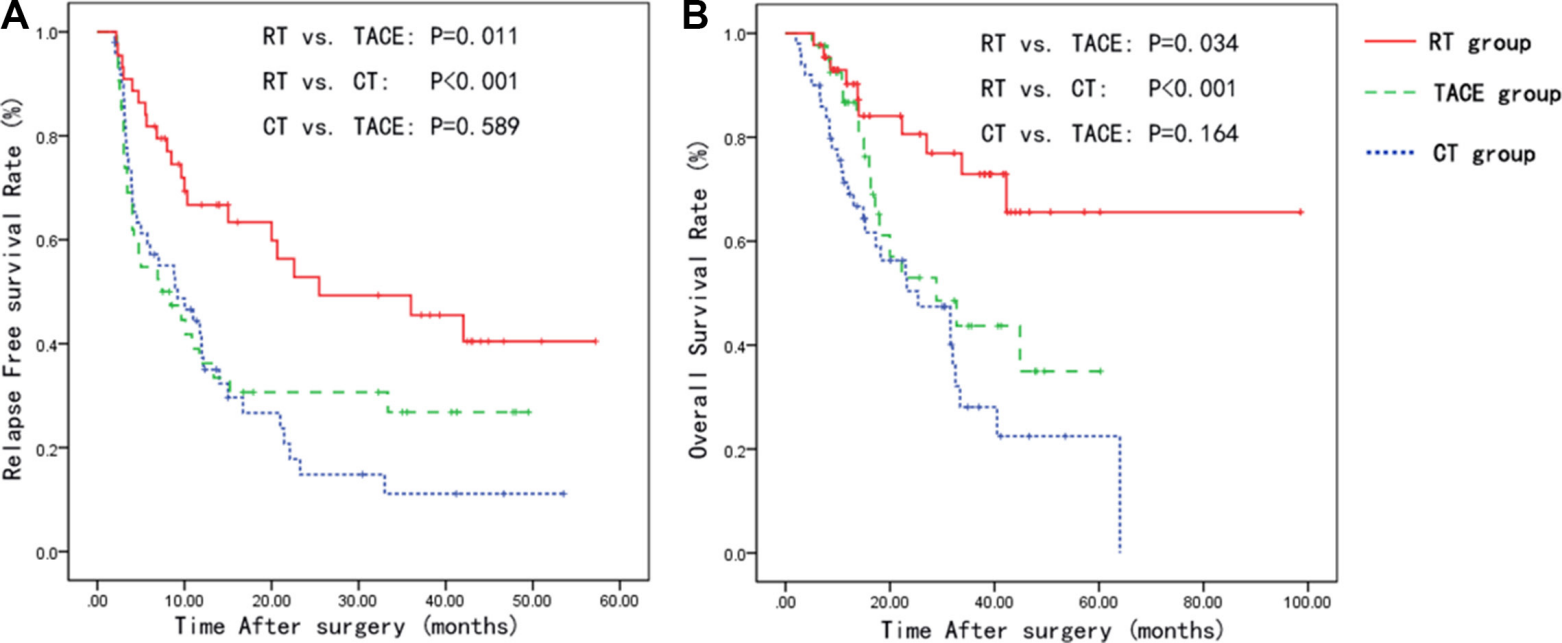
Kaplan-Meier curves for the relapse-free survival and overall survival in the CT, TACE and RT groups Abbreviations: CT: Conservative therapy; TACE: Trans catheter arterial chemoembolization; RT: Radiation therapy.

The 1-, 2-, and 3-year RFS rates were 37.4, 14.8 and 11.1% for patients in the CT group, 36.2, 30.7 and 26.8% for patients in the TACE group, 66.7, 52.8, and 45.5% for patients in the RT group, respectively. The 1-, 2-, and 3-year OS rates were 69.0, 50.4, and 28.1% for patients in the CT group, 86.7, 53.0, and 43.7% for patients in the TACE group and 90.2, 80.6, and 72.9% for patients in the RT group, respectively. The median RFS and OS periods were 9.21 and 25.37 months in the CT group, 7.41 and 28.85 months in the TACE group, and 25.47 and 72.54 months in the RT group, respectively. RT group showed a significantly longer RFS than the CT and TACE group (RT vs CT, *P* < 0.001; RT vs TACE, *P* = 0.011; Figure 1A). Likewise, patients in the RT group has longer OS compared with the CT and TACE group (RT vs CT, *P* < 0.001; RT vs TACE, *P* = 0.034; Figure 1B). However, the RFS and OS rates between the TACE and CT groups were not significant (Figure 1A and 1B). The cumulative 1-, 2-, 3-year RFS of all 136 patients were 46.5%, 32.0% and 27.0%, and their cumulative OS rates were 81.1%, 61.2% and 48.0%, respectively.

### Survival analysis according to the degree of microvascular invasion and surgical margin

The RFS and OS rates were also analyzed in the subgroups.

**Figure 2:**
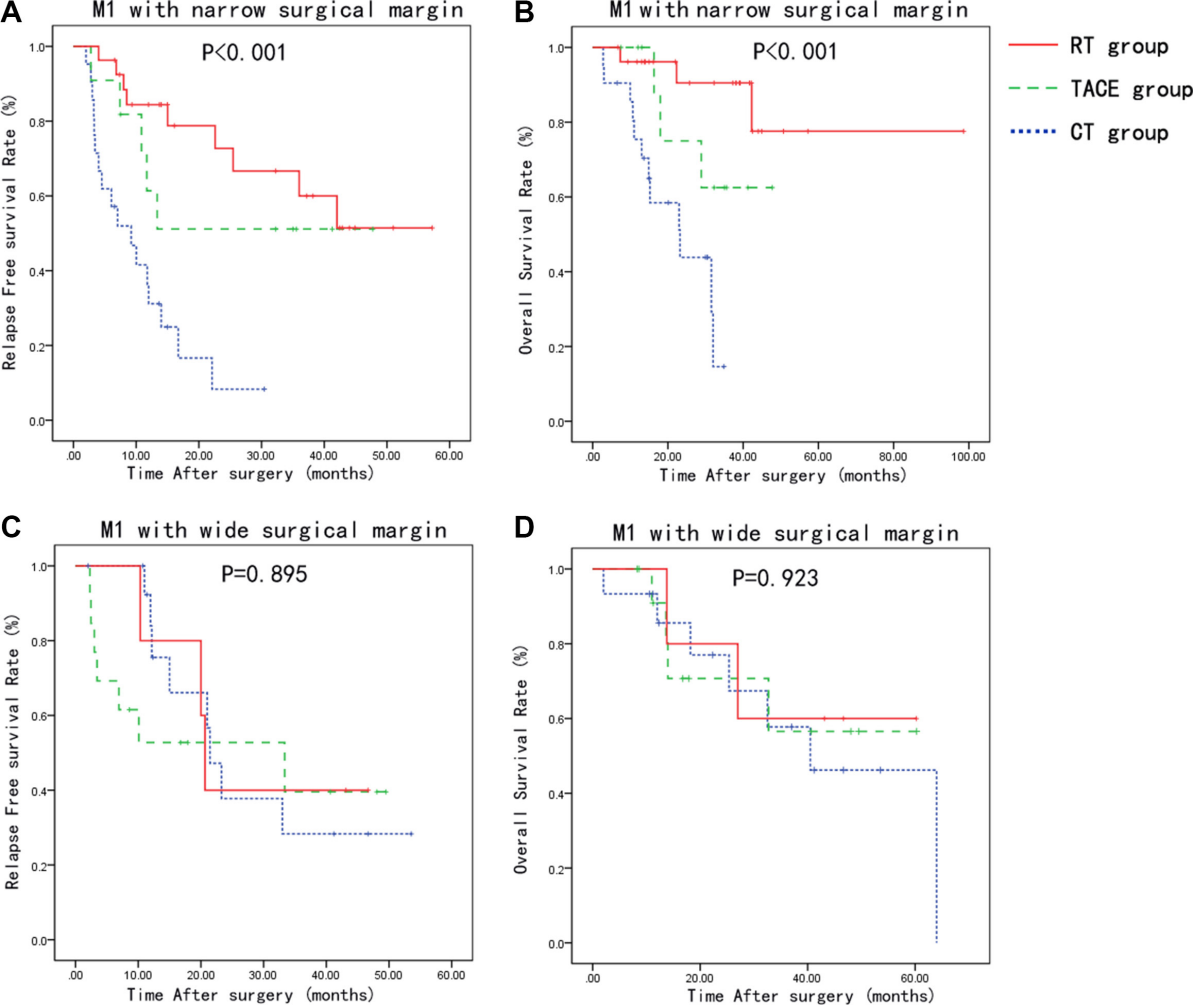
Relapse-free survival and overall survival curves of patients stratified based on the MVI type and surgical margin width Patients with low-risk MVI (M1) and narrow surgical margin has improved RFS and OS with adjuvant radiotherapy compared to CT and TACE group (**A**, **B**). There was no significant difference between the groups in the RFS and OS with low-risk MVI (M1) and wide surgical margin (**C**, **D**).

In the low-risk (M1) microvascular invasion with narrow surgical margin group, the 1-, 2-, 3-year RFS were 31.2, 8.3 and 0%, in CT group, 61.4, 51.1 and 51.1%, in TACE group, and 84.4, 51.4 and 51.4%, in RT group, respectively. The 1, 2 and 3-year OS rates were 75.4, 43.8 and 14.6% in the CT group, 87.5, 75.0 and 62.5% in the TACE group, 96.2, 90.5 and 77.6% in the RT group, respectively. RT group showed a significant longer RFS (*p* < 0.001) and OS (*p* < 0.001) than the TACE and CT groups (Figure 2A and 2B).

In the low-risk (M1) microvascular invasion with wide surgical margin group, the 1-, 2-, 3-year RFS were 75.5, 37.8 and 28.3%, in CT group, 52.7, 39.6 and 39.6%, in TACE group, and 80.0, 40.0 and 40.0%, in RT group, respectively. The 1-, 2-, 3-year OS rates were 85.6, 77.0 and 57.8% in the CT group, 90.9, 70.7 and 56.6% in TACE group, 100.0, 80.0 and 60.0% in the RT group, respectively. There was no significant difference between the groups in the RFS and OS (Figure 2C and 2D).

**Figure 3:**
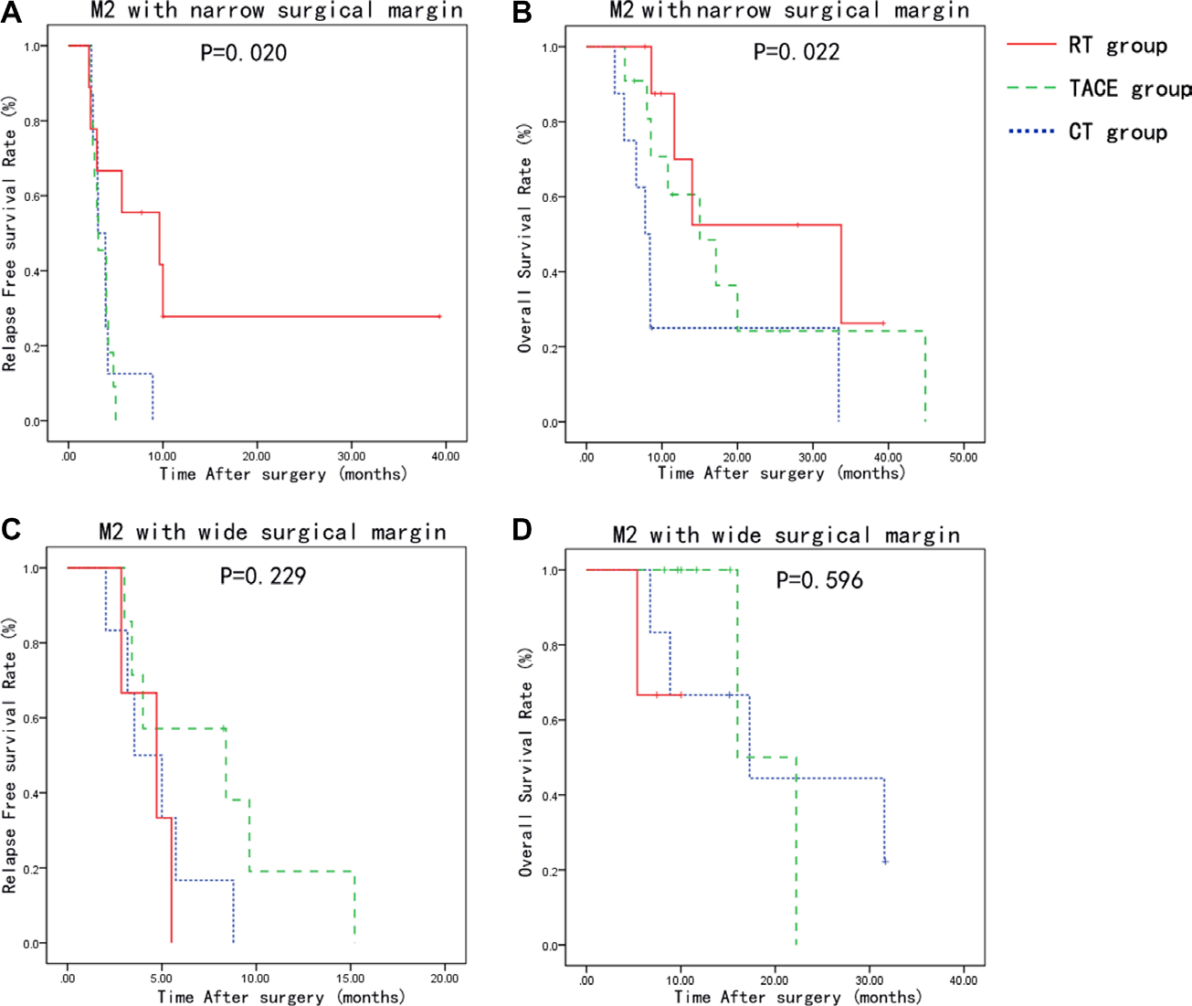
Relapse-free survival and overall survival curves of patients stratified by grade of MVI and surgical margin Patients with high-risk MVI (M2) and narrow surgical margin has improved RFS and OS with adjuvant radiotherapy compared to CT and TACE group (**A**, **B**). There was no significant difference between the groups in the RFS and OS with high-risk MVI (M2) and wide surgical margin (**C**, **D**).

In the high-risk (M2) microvascular invasion and narrow surgical margin group, the 1-, 2-, 3-year RFS rates were 0%, 0% and 0%, in CT group; 0%, 0% and 0%, and in TACE group; 27.8, 27.8 and 27.8%, in RT group, respectively. The 1-, 2-, 3-year OS rates were 25, 25 and 0% in CT group, 60.0, 24.2 and 24.2% in the TACE group, and 70.0, 52.5 and 26.2% in the RT group, respectively. The median RFS and OS period was 3.12 and 7.77 months in CT group; 3.15 and 15.00 months in TACE group; and 9.64 and 33.74 months in RT group, respectively. RT group showed a significant better RFS (*p* = 0.020) and OS (*p* = 0.022) than the other 2 groups (Figure 3A and 3B).

In the high-risk (M2) invasion and wide surgical margin group, the 1-, 2-, 3-year RFS rates were 0%, 0% and 0%, in CT group; 19.0%, 0% and 0%, and in TACE group; 0%, 0% and 0%, in RT group, respectively. The 1-, 2-, 3-year OS rates were 66.7, 44.4 and 22.2% in CT group, 100.0, 0.0 and 0.0% in the TACE group, and 66.7, 0.0 and 0.0% in the RT group, respectively. The median RFS and OS duration was 3.54 and 17.28 months in the CT group; 8.39 and 16.00 months in the TACE group; and 4.72 and 8.48 months in the RT group, respectively. There was no significant difference between the groups in the RFS and OS (Figure 3C and 3D).

### Recurrence pattern in CT, TACE and RT groups

Recurrence was observed in 89 of 136 patients. The incidence of marginal, non-marginal and extrahepatic recurrence was 4, 27 and 8 in CT group, 3, 19 and 7 in TACE group and 3, 13 and 5 in RT group, respectively. There was no significant difference in recurrent pattern between the three groups. The details of location of recurrent tumor were shown in Table 2.

**Table 2:** Pattern of recurrence in the CT, TACE and RT groups

Pattern of recurrence	Postoperative therapeutic management			*P* value
	CT (*n =* 50)	TACE (*n =* 42)	RT (*n =* 44)	
Total	39	29	21	-
Intrahepatic recurrence				0.977
Marginal	4	3	3	-
Non-marginal	27	19	13	-
Extrahepatic recurrence	8	7	5	-

### Prognostic factors affecting clinical outcomes

The univariate Cox regression analysis indicated that blood loss, tumor size, MVI classification, envelope invasion, serum AFP level, and postoperative treatment strategies as independent risk factors that influenced RFS (Table 3); while tumor size, MVI classification, envelope invasion, serum AFP level, and postoperative treatment strategies were the independent factors that influenced OS (Table 4). The multivariate analysis revealed that postoperative treatment strategies, surgical margin, envelope invasion and MVI classification were the independent prognostic factors associated with RFS (Table 3); while, postoperative treatment strategies, tumor size and MVI classification were the independent prognostic factors associated with OS (Table 4).

**Table 3:** The univariate and multivariate regression analysis to identify prognostic factors for RFS

Variable	Univariate analysis		Multivariate analysis	
	HR (95% CI)	*p* value	HR (95% CI)	*p* value
RFS				
Age	0.993 (0.975–1.010)	0.398	-	-
Gender	1.592 (0.769–3.295)	0.210	-	-
Operative time	1.001 (0.999–1.004)	0.308	-	-
Operative procedure	1.104 (0.722–1.686)	0.649	-	-
Operative method	1.108 (0.731–1.679)	0.629	-	-
Blood loss	1.000 (1.000–1.001)	0.011	-	-
Surgical margin	0.955 (0.621–1.469)	0.834	0.548 (0.343–0.875)	0.012
Tumor size	1.616 (1.085–1.243)	< 0.001	-	-
Number of tumors	1.116 (0.539–2.309)	0.768	-	-
Differentiation	1.118 (0.802–1.557)	0.512	-	-
MVI classification	5.389 (3.382–8.585)	< 0.001	5.907 (3.543–9.848)	< 0.001
Envelope invasion	3.032 (1.568–5.863)	0.001	2.868 (1.442–5.704)	0.003
Cirrhosis	1.362 (0.551–3.368)	0.503	-	-
Viral hepatitis	1.478 (0.785–2.783)	0.226	-	-
AFP level	1.813 (1.369–2.402)	< 0.001	-	-
Postoperative Treatment strategy		0.004		< 0.001
TACE	0.865 (0.534–1.402)	0.557	0.633 (0.385–1.041)	0.072
RT	0.416 (0.243–0.711)	0.001	0.278 (0.154–0.501)	< 0.001

**Table 4:** The univariate and multivariate regression analysis to identify prognostic factors for OS

Variable	Univariate analysis		Multivariate analysis	
	HR (95% CI)	*p* value	HR (95% CI)	*p* value
OS				
Age	0.993 (0.972–1.015)	0.524	-	-
Gender	1.194 (0.511–2.788)	0.682	-	-
Operative time	1.001 (0.998–1.004)	0.411	-	-
Operative procedure	1.253 (0.742–2.115)	0.400	-	-
Operative method	0.997 (0.590–1.685)	0.991	-	-
Blood loss	1.000 (1.000–1.001)	0.063	-	-
Surgical margin	0.891 (0.516–1.538)	0.678	-	-
Tumor size	1.178 (1.087–1.275)	< 0.001	1.152 (1.056–1.256)	0.001
Number of tumors	0.393 (0.096–1.614)	0.195	-	-
Differentiation	1.256 (0.812–1.944)	0.305	-	-
MVI classification	3.662 (2.130–6.297)	< 0.001	3.117 (1.771–5.486)	< 0.001
Envelope invasion	3.039 (1.213–7.617)	0.018	-	-
Cirrhosis	0.766 (0.275–2.134)	0.610	-	-
Viral hepatitis	1.596 (0.660–3.860)	0.300	-	-
AFP level	1.648 (1.173–2.315)	0.004	-	-
Postoperative Treatment strategy		0.002	-	< 0.001
TACE	0.648 (0.356–1.179)	0.155	0.423 (0.221–0.810)	0.009
RT	0.276 (0.134–0.567)	0.000	0.279 (0.136–0.574)	0.001

## DISCUSSION

Surgical resection is considered as the gold standard treatment modality for patients with preserved liver function and without portal hypertension. Unfortunately, the long-term survival after hepatectomy is not satisfactory because of the high incidence of intrahepatic tumor recurrence. MVI has been reported to be the most independent factor associated with recurrence and significantly affects the RFS and OS following curative resection or transplantation [8–10, 14]. Zhao et al. reviewed 266 patients with multinodular HCC treated with surgical resection and found that the 3-year OS rate was 16% and 58% with and without MVI, respectively [15]. Rodriguez-Peralvarez et al. performed a meta-analysis to study the prognostic impact of MVI and found that patients with MVI had significantly reduced disease-free and OS at 3- and 5-years after liver resection and transplantation [7].

### Postoperative treatment strategies

Various postoperative adjuvant therapies including TACE, radiotherapy, conservative and molecular targeted therapies are used to decrease HCC recurrence and thus prolong OS. However, the outcomes of these interventions are variable and no adjuvant therapy have been universally accepted as being effective in reducing recurrence after hepatectomy [16–21]. Moreover, studies comparing these adjuvant therapies are rare and only few studies take the MVI into consideration. The development of radiation therapy techniques, such as three-dimensional conformal or IMRT has facilitated the delivery of high radiation doses to specified target area without affecting the overall liver function [22, 23]. Wang et al. investigated the benefit of postoperative IMRT following narrow margin hepatectomy for HCC close to major vessels and found that IMRT improved 3-year overall and disease-free survival without any instance of liver damage [12]. Contrarily, the effect of adjuvant radiotherapy in patients who underwent narrow margin hepatectomy for centrally located HCC found no significant difference between the groups in the overall and recurrence-free survival at 3- and 5-years [13]. In our study, adjuvant RT showed a significantly improved RFS and OS compared to TACE and CT, which implies postoperative RT, could eliminate residual micro-metastasis-foci in the remnant liver.

The purpose of postoperative adjuvant TACE is to eliminate small intrahepatic metastases that may not have been detected preoperatively [24, 25]. But the effects of postoperative TACE and range of applications is still a debate [26]. A retrospective study with 2436 HCC patients showed that postoperative adjuvant TACE had no effect on postponing or eliminating late recurrence [27]. A meta-analysis reported that postoperative TACE significantly improved disease free survival and OS compared to control when the mean tumor size was bigger than 5 cm [28]. The superiority of adjuvant TACE over hepatectomy alone was demonstrated in another meta-analysis where postoperative TACE lessened tumor recurrence for patients with multiple nodules of > 5 cm or macrovascular invasion [29]. Nevertheless, few studies take the MVI as an independent prognosis factor into consideration to evaluate the effect of postoperative adjuvant TACE. Although, a single center retrospective study showed that postoperative adjuvant TACE may be beneficial for HCC patients with MVI; it suggested that further randomized controlled studies are needed to make a definitive conclusion [30]. In our analysis, RFS and OS was significantly shorter in patients with postoperative adjuvant TACE compared to postoperative adjuvant RT, but no significant difference between adjuvant TACE and conservative treatment was found. These results suggested that postoperative adjuvant TACE could not be effective in eliminating residual micro-metastasis-foci in the remnant liver. The possible explanations for this result could be that MVI cannot be clearly stained during the procedure of TACE, thus patients with MVI have more possibility of undetectable micro metastasis foci and TACE can’t identify and make definite elimination of residual tumor. Second, targeting to the tumor bed means extending TACE to all liver segments relative to tumor, it would severely damage liver function due to its adverse effects, and the increasing complication might increase risk of recurrence. However, the conformal or intensity modulated radiotherapy, concentrates irradiation precisely to tumor bed area and selectively spares the normal liver tissues, thus limiting the adverse effects when the tumor bed with residual micro-metastasis-foci receive sufficient radiation [12, 13].

### MVI classification and surgical margin

The feature of MVI encompasses a wide spectrum such as distance from the invaded vessel to tumor edge, the number of invaded microscopic vessels, intravascular floating tumor clusters and small vascular intratumoral spaces [31]. Each feature has different prognostic significance. Recent studies have shown that invasion of vessels ≥ 1 cm from the tumor capsule and the number of invaded vessels ≥ 5 is able to stratify patients with different risks of survival [31, 32]. MVI can be classified into two different categories that are inversely correlated with time to recurrence and survival [33]. In our study, uni- and multivariate analysis showed that MVI classification was independent factor either for RFS or OS. These results suggested that classification systems could distinguish risk features more accurately. However, since MVI disseminate mainly via portal venous branches and spread along as well as against the direction of the portal venous flow, the incidence of MVI was closely related to the distance from the tumor capsule [34]. A few studies had found that persistent residual microscopic lesion in the remnant liver tissues occur commonly around the primary tumor [23, 35–37] and MVI beyond 1 cm from the tumor capsule is very rare [31]. Thus resection margin of at least 1.0 cm is preferable to eradicate microscopic lesions and theoretically gives a higher potential for the majority of patients to reduce recurrence [38, 39]. Some studies even suggested to ensure a safe resection margin (> 2 cm) for both anatomic and non-anatomic hepatectomy [34, 40]. On the other hand, centrally located HCC lesions that adjoin main vasculatures make the wide margin hepatectomy impossible; preserving as much nontumorous liver parenchyma as possible is also an important consideration for patients with cirrhotic liver. Thus, narrow margin resection is the only option [41–44] and the addition of effectual postoperative treatment is essential clinical required.

In our study, we conducted subgroup analysis according to MVI classification and surgical margin width. For patients of narrow surgical margin, postoperative radiotherapy yield survival outcomes significantly superior than TACE or conservative treatment regardless MVI classification. Whereas, no significant difference in the survival outcomes between the three groups of patients with wide surgical margin, independent of MVI classification. Based on our data, we deduced that postoperative radiotherapy might control persistent residual microscopic lesions in the remnant liver tissue in the narrow margin patients.

### Limitations

The retrospective nature and the small sample size are the limitations of this study. However, the data of our study does provide rationale for developing a prospective study. Thus, our results should be validated in a randomized, controlled trial with large sample size to make a definitive conclusion on the advantages of adjuvant RT over adjuvant TACE and CT in HCC patients with MVI.

## MATERIALS AND METHODS

### Patients

The database of the National Cancer Center/Cancer Hospital, Chinese Academy of Medical Sciences and Peking Union Medical College was retrospectively reviewed. A total of 646 patients who underwent hepatic resection by the same team for HCC between July 2008 and December 2015 were considered for inclusion in this study. Only patients who met all of the following eligible criteria were studied: (1) primary lesion treated with curative surgical liver resection (microscopically surgical margin free of tumor); (2) MVI were proven by postoperative pathology but without macro-vascular invasion; (3) no tumor rupture and hemorrhage before and during resection; (4) no liver failure or severe complications/adverse events after surgery within 1 month; (5) no postoperative death within 3 months; (6) preoperative liver function was Child-Pugh A degree; (7) absence of previous or simultaneous malignant tumor/diseases; (8) patients with continuous follow-up records until death or censored time. The study was conducted in accordance with the principles of Good Clinical Practice and Declaration of Helsinki Guidelines. All the study protocols are approved by the ethics committee of National Cancer Center/Cancer Hospital, Chinese Academy of Medical Sciences and Peking Union Medical College. The requirement of informed consent was waived considering the retrospective nature of the study.

The 136 patients who met the above criteria were divided into 3 groups according to different postoperative treatments: (i) conservative treatment group (CT group, *n* = 50), consisting of patients treated only with supportive therapy such as nutritional and anti-HBV/HCV therapy; (ii) transcatheter arterial chemoembolization (TACE group, *n* = 42), consisting of patients treated with TACE within 2 month after surgery and supportive therapy; (iii) radiotherapy group (RT group, *n* = 44), consisting of patients treated with three-dimensional conformal or intensity modulated radiotherapy within 2 month after surgery and supportive therapy.

### Postoperative evaluation

Considering the long-standing discrepancy in definition and grading of MVI, [7] all 136 patients’ histopathological sections were retrospectively collected and reviewed by professional pathologists from our center. The pathological diagnosis and classification of MVI was based on Practice guidelines for the pathological diagnosis of primary liver cancer: 2015 update [31]. The classification of MVI is defined as follows: M0: no MVI; M1 (low-risk): MVI of < 5 and at ≤ 1 cm away from the adjacent liver tissues; and M2 (high-risk): MVI of > 5 or at > 1 cm away from the adjacent liver tissues [31]. For the subgroup analysis, the patients were stratified according to MVI classification (M1 or M2) and surgical margin width (more or less than 1 cm) into four categories: M1 with narrow surgical margin (*n* = 59), M1 with wide surgical margin (*n* = 33), M2 with narrow surgical margin (*n* = 28) and M2 with wide surgical margin (*n* = 16).

### Procedures of CT, TACE and RT

Nutritional therapy and anti-HBV/HCV therapy was given to the entire patient during postoperative period as conservative treatment to improve liver function, block the process of liver cirrhosis and prevent recurrence. Adjuvant TACE was given only once as prevention measure within two months after the operation. The Seldinger’s technique of arterial embolization was administered as standard TACE procedure. After suspicious residual tumor stain was identified, infusion of a mixture of 20–30 mg of Adriamycin and 5–10 mL of lipiodol was performed after the arteries supplying the area of tumor were catheterized superselectively. Sufficient amount of emulsion and 2–3-mm strips of Gelfoam were delivered to the suspicious residual tumor area until complete flow stagnation was achieved. For patients who had no definite residual tumor stain, TACE has been given to tumor bed area in prophylaxis. Three-dimensional conformal or intensity modulated radiotherapy plans were generated for RT procedure. The clinical treatment volume was defined as the tumor cutting bed expands a 1-cm margin, and it was added by 0.5 to 1 cm for the final planning treatment volume. The target total dose was 54–60 Gy, which was delivered by 2 Gy/fraction, 5 fractions per week.

### Follow up

After discharge from hospital, all patients were followed-up quarterly in the first 2 years and at 6-month intervals thereafter till the last follow-up (December 2016). Alpha-fetoprotein (AFP), liver function, chest X-ray, enhanced computed tomography and/or enhanced magnetic resonance imaging were performed during the follow-up visit. The HCC recurrence was diagnosed based on typical imaging findings and/or continually increased serum AFP. Biopsies were undertaken to achieve histopathology or cytopathology evidence but were not necessarily for the assessment of recurrences. The RFS and OS are the primary and secondary outcomes respectively.

RFS was defined as the time interval between the dates of surgery and the date of the first detected recurrence or censored on the date of the last follow-up. OS was recorded as time period from the date of surgery to the date of death caused by HCC recurrence or censored on the date of the last follow-up.

### Treatment for tumor recurrence

The treatment strategy for recurrence of HCC was determined by multidisciplinary team mainly based on the comprehensive consideration of tumor characteristics, liver function and general condition. Local curative treatment consisted of hepatectomy and radiofrequency ablation (RFA); regional or systemic palliative treatment, such as TACE, radiation therapy, molecular targeted therapy and chemotherapy was performed as alternative methods for recurrence treatment.

### Statistical analysis

Categorical variables were compared using Pearson Chi-square test or Fisher’s exact test as appropriate. Continuous variable are expressed as the mean ± standard deviation and compared using one-way analysis of variance (ANOVA). Univariate and multivariate Cox proportional hazards regression analysis of the clinicopathological parameters were performed to identify the independent prognostic factors to RFS and OS. The *P value*s were calculated using the Wald test. Survival analysis was conducted using the Kaplan-Meier methods, and comparisons were performed between groups and subgroups using a log-rank test. Statistical analysis was performed using IBM SPSS software (Version 19.0) and *P value* < 0.05 was considered statistically significant.

## CONCLUSIONS

In conclusion, the findings demonstrate that radiotherapy following hepatectomy could result in better survival outcomes for HCC patients with MVI than postoperative TACE or conservative treatment based on survival outcomes. This treatment strategy might especially be effective for patients with narrow surgical margin.
